# Age-related decline in cardiac autonomic function is not attenuated with increased physical activity

**DOI:** 10.18632/oncotarget.12403

**Published:** 2016-10-02

**Authors:** Hugo Njemanze, Charlotte Warren, Christopher Eggett, Guy A. MacGowan, Matthew G D Bates, Mario Siervo, Srdjan Ivkovic, Michael I. Trenell, Djordje G. Jakovljevic

**Affiliations:** ^1^ Faculty of Medical Sciences, Institute of Cellular Medicine, MoveLab, Newcastle University, Newcastle upon Tyne, UK; ^2^ MRC Centre for Ageing and Vitality, Newcastle University, UK; ^3^ Department of Cardiology, Freeman Hospital, Newcastle upon Tyne, UK; ^4^ Institute of Genetic Medicine, Newcastle University, Newcastle upon Tyne, UK; ^5^ Cardiothoracic Department, James Cook University Hospital, Middleborough, UK; ^6^ Faculty of Medical Sciences, Centre for Rehabilitation, University of Pristina, Kosovska Mitrovica, Serbia; ^7^ Clinical Research Facility, Royal Victoria Infirmary, Newcastle upon Tyne, UK

**Keywords:** ageing, cardiac autonomic function, Physical Activity, women, Gerotarget

## Abstract

Age and physical inactivity are important risk factors for cardiovascular mortality. Heart rate response to exercise (HRRE) and heart rate recovery (HRR), measures of cardiac autonomic function, are strong predictors of mortality. The present study defined the effect of age and physical activity on HRRE and HRR. Healthy women (N=72) grouped according to age (young, 20-30 years; middle, 40-50 years; and older, 65-81 years) and daily physical activity (low active <7500, high active >12,500 steps/day) performed a maximal cardiopulmonary exercise test. The HRRE was defined as an increase in heart rate from rest to 1, 3 and 5 minutes of exercise and at 1/3 of total exercise time, and HRR as the difference in heart rate between peak exercise and 1, 2, and 3 minutes later. Age was associated with a significant decline in HRRE at 1 min and 1/3 of exercise time (r= − 0.27, p=0.04, and r=−0.39, p=0.02) and HRR at 2 min and 3 min (r=−0.35, p=0.01, and r=−0.31, p=0.02). There was no significant difference in HRRE and HRR between high and low-active middle-age and older women (p>0.05). Increased level of habitual physical activity level appears to have a limited effect on age-related decline in cardiac autonomic function in women.

## INTRODUCTION

Age is the major risk factor for developing cardiovascular diseases. The number of people of the age 65 and above increases rapidly and will continue to do so in the next 20 years [1]. By 2030 approximately 20% of the population will be aged 65 and older. In this particular age group cardiac disease is the leading cause of morbidity and mortality and accounts for 40% of all deaths [1]. Aging is also associated with an increased incidence and prevalence of hypertension, coronary artery disease and heart failure [2]. Chronological age, in the absence of apparent cardiac disease, leads to concentric cardiac remodelling and a decline in diastolic function, cardiac metabolism, and maximal performance [3]. These subclinical changes may lead to cardiac pathology and increased incidence of heart failure in older age [2].

The autonomic nervous system, comprising sympathetic and parasympathetic neural outflows, combined with afferent inputs and central control mechanism plays a vital role in cardiovascular homeostasis [4]. It regulates heart rate, systemic vascular resistance and myocardial contractility [4, 5]. Measures of cardiac autonomic function also decline with age, including heart rate response to exercise and heart rate recovery following exercise, which have been shown to be strong predictors of morbidity and mortality in healthy individuals and those with cardiac diseases [6-10].

Higher levels of physical activity are associated with a 30-40% reduced rate of all-cause and cardiovascular mortality in both women and men [11-14]. The most recent data from the Women's Health Initiative study suggest that low active and sedentary women had 63% greater risk of developing cardiovascular disease than high active women [15]. A recent study in women also suggests that higher levels of habitual physical activity preserve cardiac metabolism and exercise capacity with ageing [3]. Exercise training improves cardiac autonomic regulation of the heart in both healthy people and those with cardiac disorders [16-19]. However, limited evidence is available on the effect of objectively evaluated habitual daily physical activity (as distinct from exercise training, *per se*) on the age-related changes in cardiac autonomic regulation and heart rate response to exercise (HRRE) and heart rate recovery (HRR) following exercise. Due to a significant difference in cardiac ageing between men and women previously described (2-5), the present study was designed to define the effect of habitual daily (ambulatory) physical activity on age-related changes in cardiac autonomic function represented by HRRE and HRR in women. We hypothesized that a high level of daily physical activity (i.e. >12.500 steps per day) will attenuate age-related changes in cardiac autonomic function in women.

## RESULTS

Out of the 129 women who were contacted by the research team, a total of 72 met the study inclusion criteria and were further stratified according to daily physical activity into low- or high-active group. In terms of intensity, the level of physical activity ranged from low to moderate (≤6 metabolic equivalent units), which was expected as none of the participants were taking part in regular exercise.

**Figure 1:**
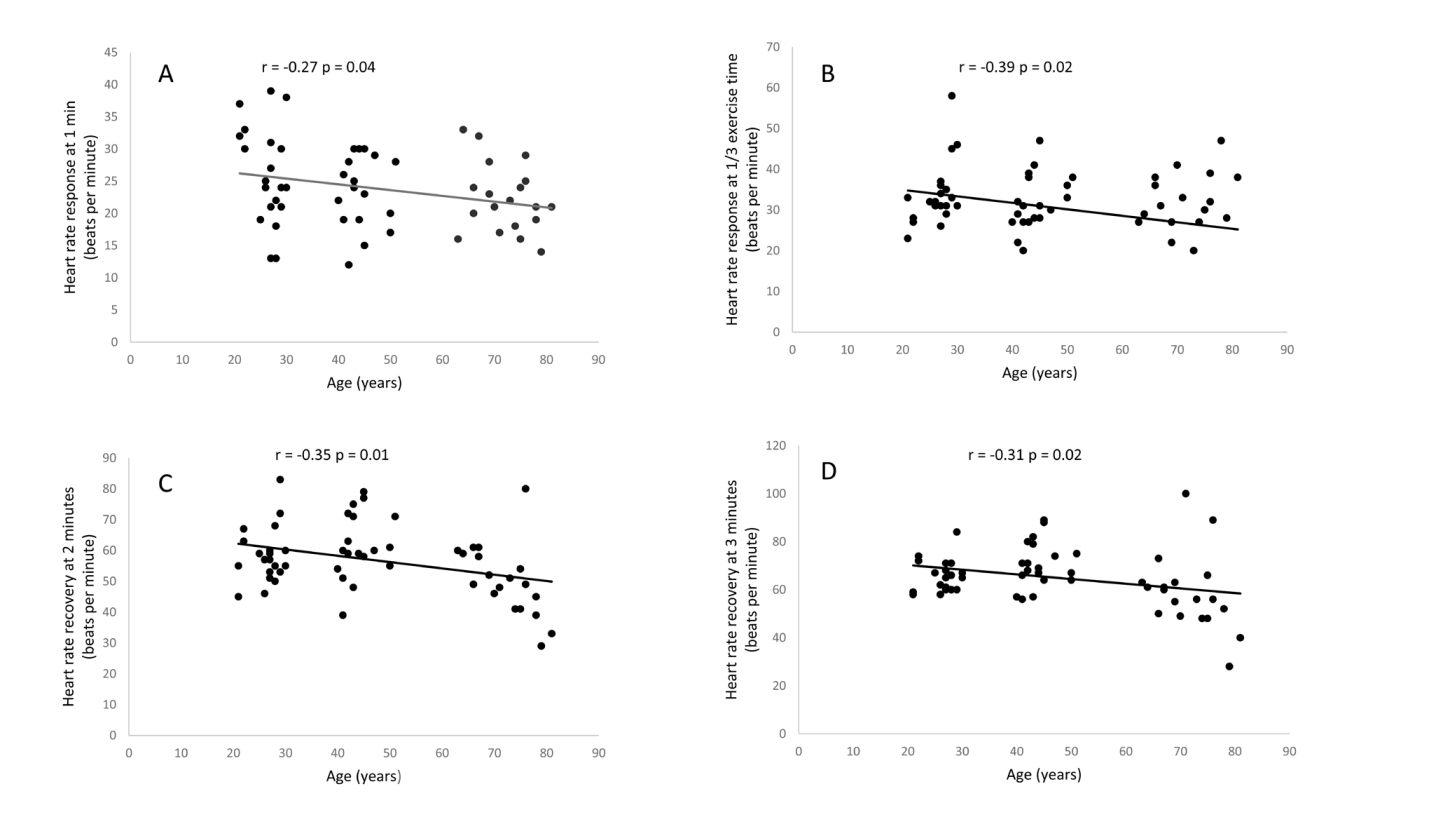
Relationship between age and heart rate response at one minute (**A**), heart rate response at 1/3 exercise time (**B**), heart rate recovery at two minutes (**C**) and heart rate recovery at 3 minutes (**D**)

Table 1 shows demographic details, resting, and exercise cardiovascular and metabolic measures in different age groups. There was a mild to moderate significant relationship between the age HRRE and HRR (Figure 1), with older women demonstrating significantly lower HRRE at 1/3 exercise time (p<0.05, Table 2). HRR at two minutes following exercise was significantly lower in older group (p<0.05, Table 2).

**Table 1:** Demographic details, resting, and exercise cardiovascular and metabolic measures in different age groups

	Young	Middle-age	Older
***Demographics***			
Age, y	22±3**	45±3†	72±5*
Weight, Kg	66.0±15.0	69.5±11.9	66.6±10.7
Height, m	1.65±0.06†	1.67±0.05†	1.59±0.05
Body mass index, Kg/m^2^	24±5	25±4	26±3
***Resting metabolic and cardiac variables***			
Oxygen consumption, L/min	0.25±0.05	0.25±0.04	0.24±0.03
Respiratory exchange ratio	0.89±0.16	0.91±0.07	0.92±0.11
Heart rate, bpm	76±11	71±10	70±8
Stroke Volume, mL/beat	88.7±15.5†	88.3±16.3†	73.1±14.0
Cardiac power output, watts	1.29±0.30	1.39±0.34	1.17±0.21
Mean arterial blood pressure, mmHg	89±8	100±10*	104±10*
Cardiac output, L/min	6.5±1.1†	6.3±1.3†	5.1±0.8
Systemic vascular resistance dyne/(s-cm^5^)	1118±187†	1316±278†	1693±344
Fasting glucose, mmol/L	5.1±0.5	4.6±0.3	5.3±0.5
Total cholesterol, mmol/L	4.4±0.9	4.4±1.7	5.5±0.8
Triglycerides, mmol/L	0.8±0.3	0.9±0.5	1.2±0.6
***Peak exercise metabolic and cardiac variables***			
Oxygen consumption, L/min	1.85±0.32†	1.96±0.44†	1.37±0.23
Oxygen consumption, ml/kg/min	29.37±6.44†	28.69±8.41†	21.0±4.38
Respiratory exchange ratio	1.17±0.07	1.20±0.08	1.17±0.11
Heart rate, bpm	178±11.83†	170±10.15†	140±13.65
Stroke Volume, mL	87.3±16.2	92.7±19.32	87.0±17.0
Cardiac Output L/min	15.4±2.6†	15.4±2.9†	12.1±2.3
Cardiac power output, watts	3.83±0.84†	4.13±0.78†	3.38±0.71
Mean arterial pressure, mmHg	111.6±11.71	121.6±10.7*	125.8±9.22*
Systemic vascular resistance dyne/(s-cm^5^)	586±110†	654±113†	862±180

* significant difference from young (p<0.05)

** significant difference from middle (p<0.05)

† significant difference from old (P<0.05)

**Table 2:** Effect of age on heart rate response to exercise and recovery

	Young	Middle-Age	Older
***Heart Rate Response to Exercise (beats/min)***			
At one minute	26±8	23±6	22±5
At three minutes	24±7	23±7	26±7
At five minutes	32±7	30±6	31±8
At 1/3 exercise time	34±8*	32±7	26.1±7.4
***Heart Rate Recovery (beats/min)***			
At one minute	44±15	46±15	38±10
At two minutes	58±9*	62±10*	50±11
At three minutes	66±7*	71±10*	59±16

* significant difference from old (p<0.05)

Table 3 shows cardiac and metabolic variables stratified by age and physical activity. At peak exercise there were no significant differences in cardiac output, mean arterial blood pressure and cardiac power output between very active and low active groups. However, there was a significant difference in peak oxygen consumption between high and low active groups (p<0.05). Interestingly, in contrast to young-age group, physical activity had no effect on measures of cardiac autonomic function in middle- and older-age groups (Table 4).

**Table 3:** Participant characteristics, resting and peak exercise cardiac and metabolic variables stratified by age and physical activity

	Young		Middle-age		Older	
	High active	Low active	High active	Low active	High active	Low active
***Demographics***						
Age, y	28±1	25±3	44±3	45±4	70±5	74±5
Weight, kg	59.7±7.5	73±18.2*	67.5±13.7	71.7±9.6	60.3±7.7	72.9±9.8*
Height, m	1.67±0.06	1.60±0.07	1.67±0.05	1.66±0.06	1.57±0.06	1.60±0.04
***Resting metabolic and cardiac variables***						
Oxygen consumption L/min	0.27±0.07	0.24±0.10	0.27±0.05	0.24±0.03	0.23±0.03	0.25±0.02
Oxygen consumption, ml/kg/min	4.79±1.20	3.4±1.15*	4.10±0.90	3.31±0.54*	3.86±0.50	3.40±0.36*
Respiratory exchange ratio	0.94±0.11	0.87±0.2	0.90±0.06	0.91±0.07	0.90±0.1	0.90±0.11
Heart rate, bpm	73±12	78±10	70±13	71±10	71±8	70±9
Stroke volume, mL/heart beat	88±13	90±17	90±15	86±16	64±13	82±9*
Cardiac power output, watts	1.26±0.34	1.31±0.20	1.37±0.34	1.42±0.34	1.06±0.20	1.30±0.14
Mean arterial pressure, mmHg	89±8	89±9	97±11	102±10	106±12	102±9
Cardiac output, L/min	6.4±1.2	6.7±1.0	6.3±1.3	6.2±1.3	4.5±0.7	5.6±0.5*
Systemic vascular resistance dyne/(s-cm^5^)	1146±194	1086±183	1275±256	1366±309	1751±305	1500±191*
***Peak exercise metabolic and cardiac variables***						
Oxygen consumption, L/min	2.05±0.24	1.70±0.3*	2.24±0.39	1.62±0.44**	1.45±0.3	1.3±0.14
Oxygen consumption, ml/kg/min	34.6±3.7	23.6±2.8**	34.1±7.7	22.2±2.4**	24.1±3.8	17.8±2.1**
Respiratory exchange ratio	1.17±0.08	1.16±0.1	1.17±0.07	1.24±0.08	1.19±0.1	1.2±0.10
Heart rate, bpm	177±13	178±10	172±8	167±12	145±11	134±14
Stroke Volume, mL	94±14	83±18	96±20	89±19	83±19	91±15
Cardiac Output L/min	16.5±2.1	14.7±2.8	16.2±2.9	14.4±2.7	12.0±2.4	12.2±2.4
Cardiac power output, watts	4.00±0.71	3.73±0.90	4.24±0.72	4.00 ±0.86	3.40±0.70	3.36±0.77
Mean arterial pressure, mmHg	110±12	114±11	119±12	125±8	128±8	124±10
Systemic vascular resistance dyne/(s-cm^5^)	542±101	633±104	604±120	715±128	805±198	840±167

* significantly different from high active group (p<0.05);

** significantly different from high active group (p<0.01)

**Table 4:** Heart rate response to exercise and recovery stratified by age and physical activity

	Young		Middle-age		Older	
	High active	Low active	High active	Low active	High active	Low active
***Heart Rate Response to Exercise (beats/min)***						
At one minute	22±5	30±8*	22±7	23±4	23±6	21±5
At three minutes	20±6	28±6**	21±7	25±6	25±6	26±9
At five minutes	30±8	35±6	29±5	32±7	31±9	31±6
At 1/3 exercise time	36±9	32±7	33±8	30±5	26±6	26±9
***Heart Rate Recovery (beats/min)***						
At one minute	48±12	40±8	46±10	47±12	40±6	36±12
At two minutes	62±10	55±7	63±10	60±12	53±8	48±14
At three minutes	67±7	65±6	71±11	71±9	63±15	54±17

* significantly different from high active group (p<0.05);

** significantly different from high active group (p<0.01)

When data were combined from all age groups and activity levels, there were no significant relationships between HRR at one minute and maximum cardiac power output (r= 0.17 p= 0.21) or maximum stroke volume (r= 0.12; p= 0.35). There was also no significant relationship between HRR at one minute and maximum oxygen consumption (r= 0.23; p= 0.07) or maximum work rate (r= 0.26; p= 0.05).

**Figure 2:**
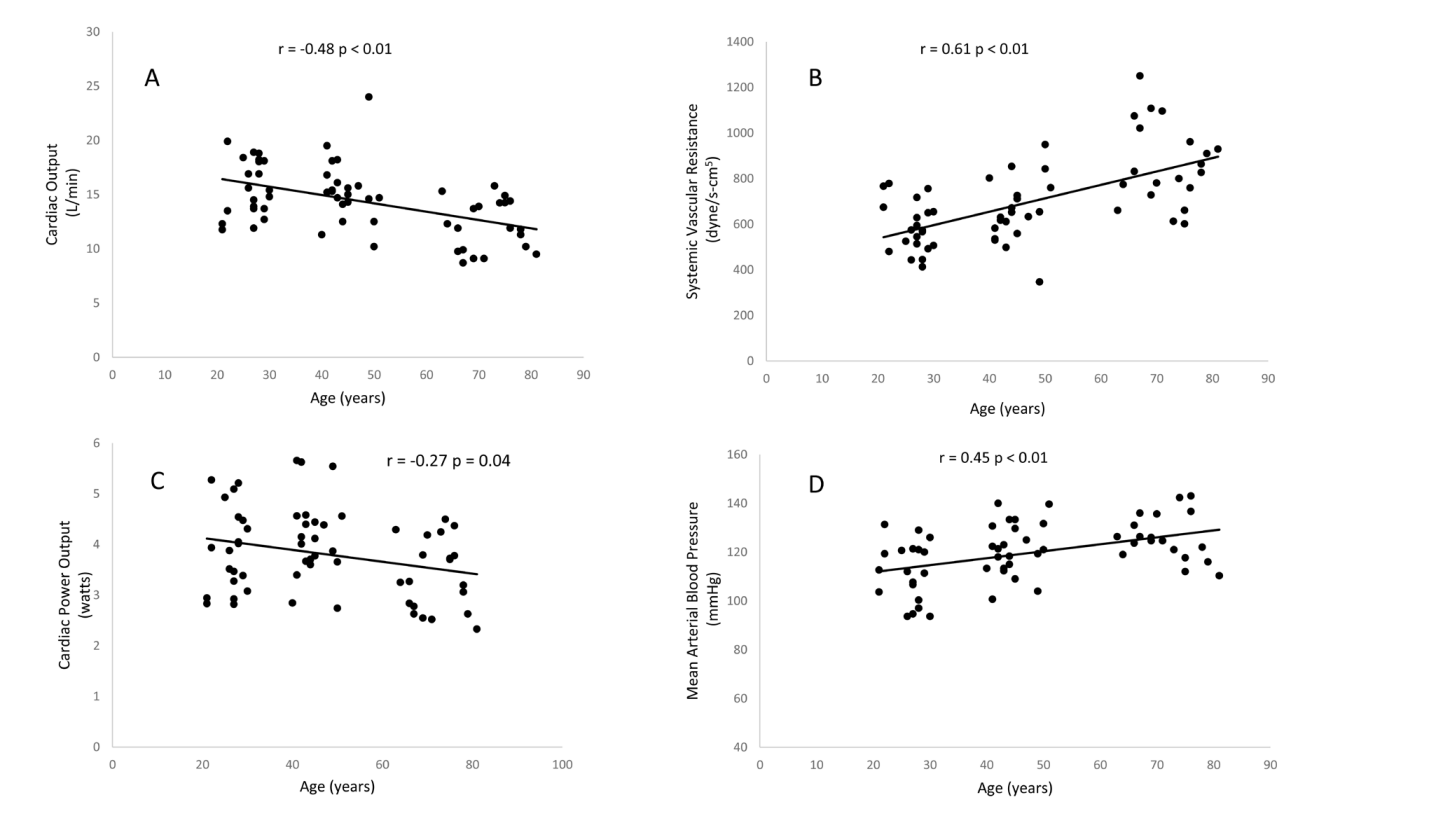
Relationship between age and maximum cardiac output (**A**), maximum systemic vascular resistance (**B**), maximum cardiac power output (**C**), maximum mean arterial pressure (**D**)

## DISCUSSION

Heart rate response to exercise and recovery is an established measure of cardiac autonomic function and strong indicator of all-cause and cardiovascular mortality [7-10]. The present study is the first to evaluate the effect of objectively measured daily physical activity on age related changes in autonomic function, as assessed by HRRE and HRR. The major findings suggest that ageing is associated with a significant decline in cardiac autonomic function, and increased levels of daily physical activity have limited effect on this age-related decline in heart rate response to exercise and recovery.

Our finding that aging in the absence of cardiovascular and other chronic diseases leads to decline in cardiac autonomic function is in agreement with those of previous studies [20-22]. It has been suggested that age-related decline in cardiac vagal modulation is a result of a significant decline in cardiac parasympathetic activity [23]. The cause for the decline in autonomic control of the heart is thought to be a result deterioration of cardiovagal baroreflex sensitivity [23]. One of the primary mechanisms believed to be responsible for such change is decline in arterial compliance (i.e. in carotid artery and aorta) which determines the amount of stretch to which baroreceptors are exposed for any change in intravascular pressure [8, 23]. Alterations in muscarinic receptors along with their signalling pathway has been considered a potential contributory factor in reduced baroreflex sensitivity with ageing [4].

The novel finding in the present study is that a high level of daily habitual physical activity (>12,500 steps/day) had no effect on heart rate response to exercise and recovery in middle- and older-age women. This is to some extent surprising considering the well-defined benefits of a physically active lifestyle on overall cardiovascular morbidity and mortality in both women and men [11-14]. In contrast to our study, which evaluated the effect of habitual (daily) physical activity in healthy women, many other studies evaluated the effect of exercise on cardiac autonomic regulation. Results of these studies suggest the benefits of exercise training on measures of cardiac autonomic function [16-19, 24-27]. It was postulated that the intensity of exercise in addition to its volume may play an important role in exercise-induced changes in cardiac autonomic regulation [27, 28]. In our study, the intensity level of physical activity that participants demonstrated (i.e. <6 metabolic equivalent units, likely represented by slow walking) was perhaps lower than a standard aerobic or high intensity interval exercise training, or even brisk walking recommended by the public health authorities. This may further suggest that potentially there are specific requirements in the intensity and duration of physical activity and exercise in order to induce beneficial changes in cardiac autonomic function and that increased level of daily (habitual) physical activity (e.g. slow walking) may not be sufficient to attenuate decline in cardiac autonomic function with ageing. A previous study also reported that recreational endurance athletes demonstrate significantly greater sympathetic modulation compared to the general population [29], further suggesting that chronic exposure to exercise may be required for potentially greater benefits to cardiac autonomic function.

In contrast with cardiac autonomic regulation, the data demonstrate that cardiorespiratory fitness (aerobic capacity), represented by peak oxygen consumption and exercise performance (work rate), and were markedly influenced by daily activity. One of the physiological mechanisms that may explain such finding is that anaerobic threshold point occurs significantly earlier in low active than in those highly active women. As the rate of lactate acid production and accumulation exceeds its removal from the muscle, it is likely to cause an onset of fatigue and consequent termination of the exercise test earlier, resulting in lower exercise performance (work rate) in inactive women.

The present study is not without limitations. All subjects in this study were female and as previously stated, there are physiological gender differences in cardiovascular adaptations to aging and physical activity. All of the women who took part in the study were Caucasian, therefore it is not known whether these results are applicable to women of a different ethnicity. Study participants were also not followed longitudinally to observe any potential changes in cardiac autonomic functions that may occur with further ageing.

In conclusion, the present study is the first to demonstrate the effect of objectively assessed daily physical activity on age-related changes in cardiac autonomic function. Our results confirm that aging is associated with a significant decline in cardiac autonomic function, and provide novel evidence that high level of daily physical activity has a limited effect on age-related decline in cardiac autonomic function. Further investigations are warranted to develop effective interventions to improve cardiac autonomic function in older age.

## MATERIALS AND METHODS

In this prospective, observational, single-centre study 72 healthy women were recruited from the Newcastle upon Tyne area (United Kingdom) into three age groups: young- (ages 20-30 years, n=24), middle- (ages 40-50 years, n=24), and older-age (65-81 years, n=24) women. Subjects were included into the study if their objectively evaluated average daily physical activity level fell into a low active i.e. <7,500 steps/day group or a high active group i.e. >12,500 steps/day, as previously suggested [30]. Participants were not stratified according to their workplace activity bur rather overall physical activity levels but with no intention participating in an exercise programme or training regime. Only subjects with normal fasting glucose and lipid profile, body mass index <30 kg/m^2^; normal resting blood pressure and electrocardiogram and able to undertake a maximal graded cardiopulmonary exercise stress test were included. Subjects were excluded if they: (i) had any prior history of cardiovascular or chronic pulmonary disease, diabetes, or were using medication known to effect cardiovascular function, current or previous smoking or exercise-limiting orthopaedic impairment; (ii) performed regular exercise (≥2 times week) over the previous three years or had been professional or semi-professional athletes; (iii) performed an average daily number of steps between 7,500 and 12,500 steps/day. All subjects signed an informed consent form and the study was approved by the NHS Sunderland Research Ethics Committee.

Physical activity was assessed objectively using a validated portable multi-sensor array (Sensewear Pro3, Bodymedia Inc, PA, USA) [31]. The monitor was worn for seven days and was only removed for bathing. Additionally, self-reported physical activity measurement was assessed using the International Physical Activity Questionnaire [32]. All subjects performed a maximal graded cardiopulmonary exercise test using an electro-magnetically controlled semi-recumbent cycle ergometer (Corival, Lode, Groningen, Netherlands) with online gas exchange measurements (Metalyzer 3B, Cortex, Leipzig, Germany) and non-invasive central hemodymamics by the bioreactance method (NICOM, Cheetah Medical, Deleware, USA), as previously described [33-35]. The maximal progressive exercise test included cycling at 20 watts for 3 minutes followed by 10-watt increments every minute until volitional exhaustion. The 12-lead ECG (Custo, CustoMed GmbH, Ottobrunn, Germany) was continuously monitored and blood pressure (Tango, SunTech Medical, Morrisville, NS, USA) recorded at rest, during exercise and recovery. The test was terminated when the subject was unable to pedal at a cadence of 50 revolutions per minute or they reached maximal oxygen consumption i.e. no further increase in oxygen uptake despite increase in work load. Peak oxygen consumption was defined as the average oxygen uptake during the last minute of exercise. Cardiac power output (expressed in watts), a measure of overall cardiac function and pumping capability, was calculated as the product of cardiac output and mean arterial blood pressure [3].

Prior to graded exercise testing, resting heart rate (HR) was calculated as the mean HR during the last five minutes of the 10 minute supine rest. The change in HR at one minute of exercise was calculated as the difference between HR at one minute of exercise and immediately prior to commencing exercise [8]. The change in HR at three minutes of exercise was calculated as the differences between HR at three minutes of exercise and immediately prior to commencing exercise. The change in HR at five minutes of exercise was calculated as the differences between HR at five minutes exercise and immediately prior to commencing exercise. The change in HR at 1/3 exercise time was calculated as the difference between HR immediately prior to commencing exercise and the HR at 1/3 of the completed exercise time. Once graded cardiopulmonary exercise testing was completed, participants undertook a 10 minute seated recovery during which HR was recorded. Heart rate recovery (HRR) was calculated as the difference between peak HR and HR recorded one, two and three minutes post completion of the graded cardiopulmonary exercise testing [8].

All statistical analysis was carried out using SPSS version 19.0 (SPSS inc. Chicago, Illinois, USA). Prior to statistical analysis, data were checked for univariate and multivariate outliers using standard Z-distribution cut-offs and Mahalanobis distance tests. Normality of distribution was assessed using a Kolmogorov-Smirnov test. Two-way analysis of variance (ANOVA) was used to determine the main effects of age and physical activity and the interaction between the age and physical activity. The difference between low active and high active women within the same age group was identified using independent t-tests or Mann Whitney U test, as appropriate with Bonferroni correction for multiple comparisons. Pearson's or Spearman's coefficient of correlation was used to assess the relationship between the age and measures of cardiac autonomic function variables, as appropriate. Statistical significance was indicated if P<0.05. All data are presented as mean±SD unless otherwise indicated.
